# Supplementary material for: Lipolysis regulates major transcriptional programs in brown adipocytes
Source: Nat Commun. 2022 Jul 8;13:3956. doi: 10.1038/s41467-022-31525-8 (PMC9270495; doi:10.1038/s41467-022-31525-8)
Supplement: Supplementary file 1 — Supplementary Information [file 41467_2022_31525_MOESM1_ESM.pdf]

Lipolysis regulates major transcriptional programs in brown adipocytes

Supplementary information

Supplementary Figure 1: Knockdown of ABHD5 abrogates SR-3420-activated gene expression

Supplementary Figure 2: Knockdown of PPARs

Supplementary Figure 3: Epigenomic response to SR-3420

Supplementary Figure 4: Atglistatin does not override the intrinsic rhythmicity in WAT and liver

Supplementary Note : Codes for STAR and MACS2

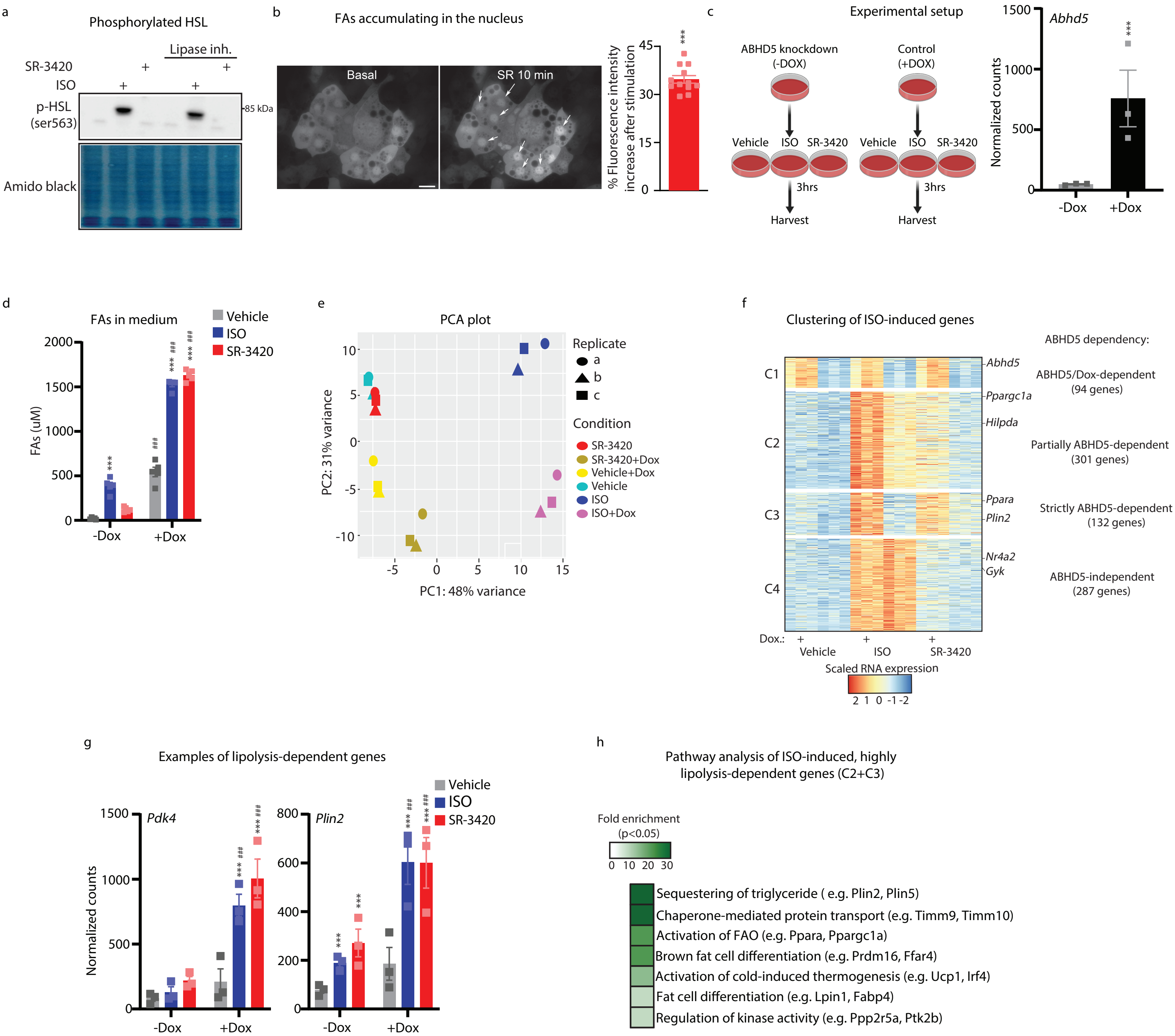
a
Phosphorylated HSL
Lipase inh.
SR-3420
ISO
p-HSL (ser563)
85 kDa
Amido black
b
FAs accumulating in the nucleus
Basal
SR 10 min
% Fluorescence intensity increase after stimulation
c
Experimental setup
ABHD5 knockdown (-DOX)
Control (+DOX)
Vehicle ISO SR-3420
3hrs
Harvest
Abhd5
Normalized counts
-Dox +Dox
d
FAs in medium
Vehicle
ISO
SR-3420
FAs (uM)
e
PCA plot
Replicate
a
b
c
Condition
SR-3420
SR-3420+Dox
Vehicle+Dox
Vehicle
ISO
ISO+Dox
PC2: 31% variance
PC1: 48% variance
f
Clustering of ISO-induced genes
ABHD5 dependency:
C1
C2
C3
C4
Abhd5
Ppargc1a
Hilpda
Ppara
Plin2
Nr4a2
Gyk
ABHD5/Dox-dependent (94 genes)
Partially ABHD5-dependent (301 genes)
Strictly ABHD5-dependent (132 genes)
ABHD5-independent (287 genes)
Dox.:
Vehicle ISO SR-3420
Scaled RNA expression
2 1 0 -1 -2
g
Examples of lipolysis-dependent genes
Pdk4
Plin2
Normalized counts
Vehicle
ISO
SR-3420
-Dox +Dox
h
Pathway analysis of ISO-induced, highly lipolysis-dependent genes (C2+C3)
Fold enrichment (p<0.05)
0 10 20 30
Sequestering of triglyceride (e.g. Plin2, Plin5)
Chaperone-mediated protein transport (e.g. Timm9, Timm10)
Activation of FAO (e.g. Ppara, Ppargc1a)
Brown fat cell differentiation (e.g. Prdm16, Ffar4)
Activation of cold-induced thermogenesis (e.g. Ucp1, Irf4)
Fat cell differentiation (e.g. Lpin1, Fabp4)
Regulation of kinase activity (e.g. Ppp2r5a, Ptk2b)

Supplementary Figure 1: Knockdown of ABHD5 abrogates SR-3420-activated gene expression

(Related to Figure 1)

Mouse immortalized *in vitro* differentiated brown adipocytes with stable knockdown of ABHD5 or ectopic expression of EYFP-SRC1. Six days after preadipocyte differentiation, cells were incubated with differentiation medium either in the presence (+Dox) or absence (-Dox) of doxycycline (1 µg/mL) for 24 hours to induce *Abhd5*. Cells were treated with either vehicle, 10 nM isoproterenol (ISO) (blue bar), or 20 µM SR-3420 (red bar) for 3 hrs before harvest.

a Western blot showing levels of phosphorylated HSL (phosphor Ser563) in differentiated mouse brown adipocytes. n=1 biologically independent experiments examined **b** Quantification of nuclear EYFP-SRC1 in mouse brown adipocytes following stimulation SR-4995 for 10 min. Confocal fluorescence images were captured at under basal conditions or 10 minutes after stimulation with SR-4995 (10µM). n=12. **c** Schematic overview of experimental setup and mRNA expression of *Abhd5* quantified by qPCR. Created with BioRender.com. n=3 biologically independent experiments examined, each carried out in a technical duplicate. Scale bar denotes 10 µM **d** Basal and stimulated FA release into culture medium 3 hours following treatment with either vehicle, ISO, or SR-3420. n=5 biologically independent experiments examined, each carried out in a technical duplicate **e, f** PCA plot and heatmap from RNA-seq data showing K-means clustering (ISO/SR-3420 vs. vehicle: Log2FC≤ -1 or Log2FC≥1, pAdj<0.05) in differentiated mouse brown preadipocytes. n=3 biologically independent experiments examined, each carried out in a technical duplicate **g** mRNA expression pattern of representative lipolysis-activated genes from Supplementary Figure 1e. n=3 biologically independent experiments examined, each carried out in a technical duplicate. **h** Pathways significantly enriched (FDR<0.05) among lipolysis-activated genes in Cluster 2 and Cluster 3 from Supplementary Figure 1e.

For all panels, error bars represent +/- SEM of 3-5 independent biological experiments. Statistical significance was determined by DESeq2 using FDR/Benjamini-Hochberg correction for Supplementary Figure 1c and Supplementary Figure 1g and by one-way ANOVA with Tukey's multiple comparisons test for Supplementary Figure 1d and paired Student's t-test for Supplementary Figure 1b (p≤0.05 = *, p≤0.01 = **, p≤0.001 = ***). * versus Vehicle (+/- Dox), # versus -Dox.

a

PPARa knockdown

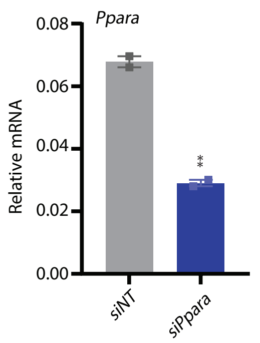

b

Volcano plots

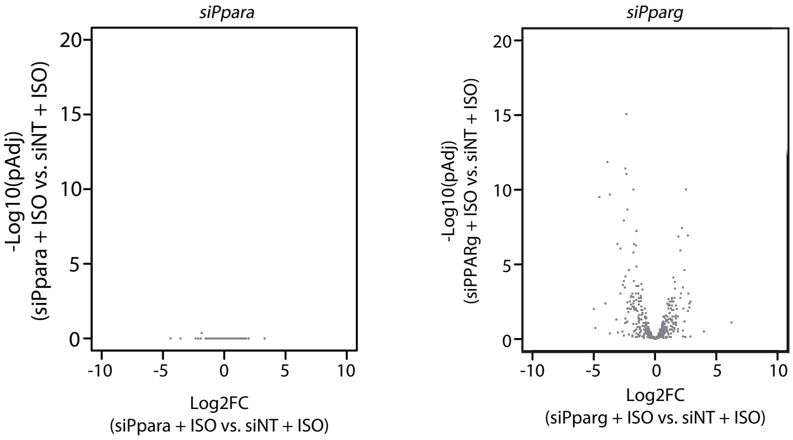

Supplementary Figure 2: Knockdown of PPARs

(Related to Figure 3)

Mouse *in vitro* differentiated brown adipocytes were treated with siRNA against *Pparg*, *Ppara* or *NT* (negative control) for 3 days and subsequently with 100nM isoproterenol (ISO) for 3 hrs before harvest.
a mRNA expression of *Ppara* with or without *Ppara* knockdown in mature brown adipocytes quantified using qPCR. Error bars represent +/- SEM of 3 independent biological experiments. Statistical significance was determined by unpaired Student's t-test ($p \leq 0.05$ = *, $p \leq 0.01$ = **, $p \leq 0.001$ = ***) **b** Volcano plots of the effect on lipolysis-activated genes (C1+C3+C4 from Fig. 1d) in mouse brown adipocytes stimulated with 100nM ISO with or without knockdown of *Ppara* or *Pparg*.

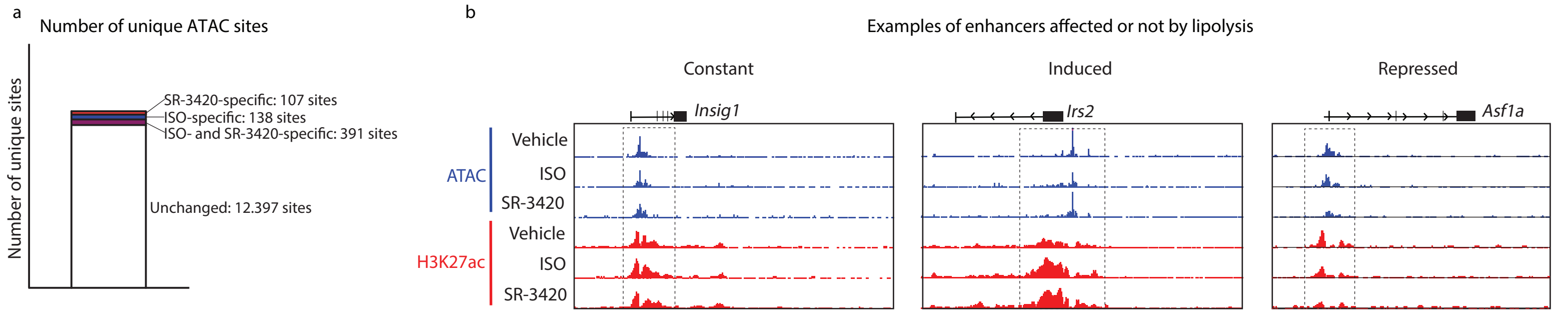
a
Number of unique ATAC sites
Number of unique sites
SR-3420-specific: 107 sites
ISO-specific: 138 sites
ISO- and SR-3420-specific: 391 sites
Unchanged: 12.397 sites
b
Examples of enhancers affected or not by lipolysis
Constant
Induced
Repressed
Insig1
Irs2
Asf1a
ATAC
H3K27ac
Vehicle
ISO
SR-3420
Vehicle
ISO
SR-3420

Supplementary Figure 3: Epigenomic response to SR-3420

(Related to Figure 4)

Mouse *in vitro* differentiated brown adipocytes were pre-treated with 10µM Atglistatin (ATGL inhibitor) and 20µM CAY10499 (HSL inhibitor) for 1h and subsequently with 100nM isoproterenol (ISO) or 20µM SR-3420 for 3 hrs before harvest for ChIP and ATAC.

a Number of chromatin regions defined by ATAC-seq (FDR<0.05)) with differential accessibility between vehicle, ISO and C162 stimulated cells for 3 hrs. **b** UCSC Genome Browser screenshots of H3K27ac and ATAC signal at representative gene loci of constant, induced and repressed enhancers.

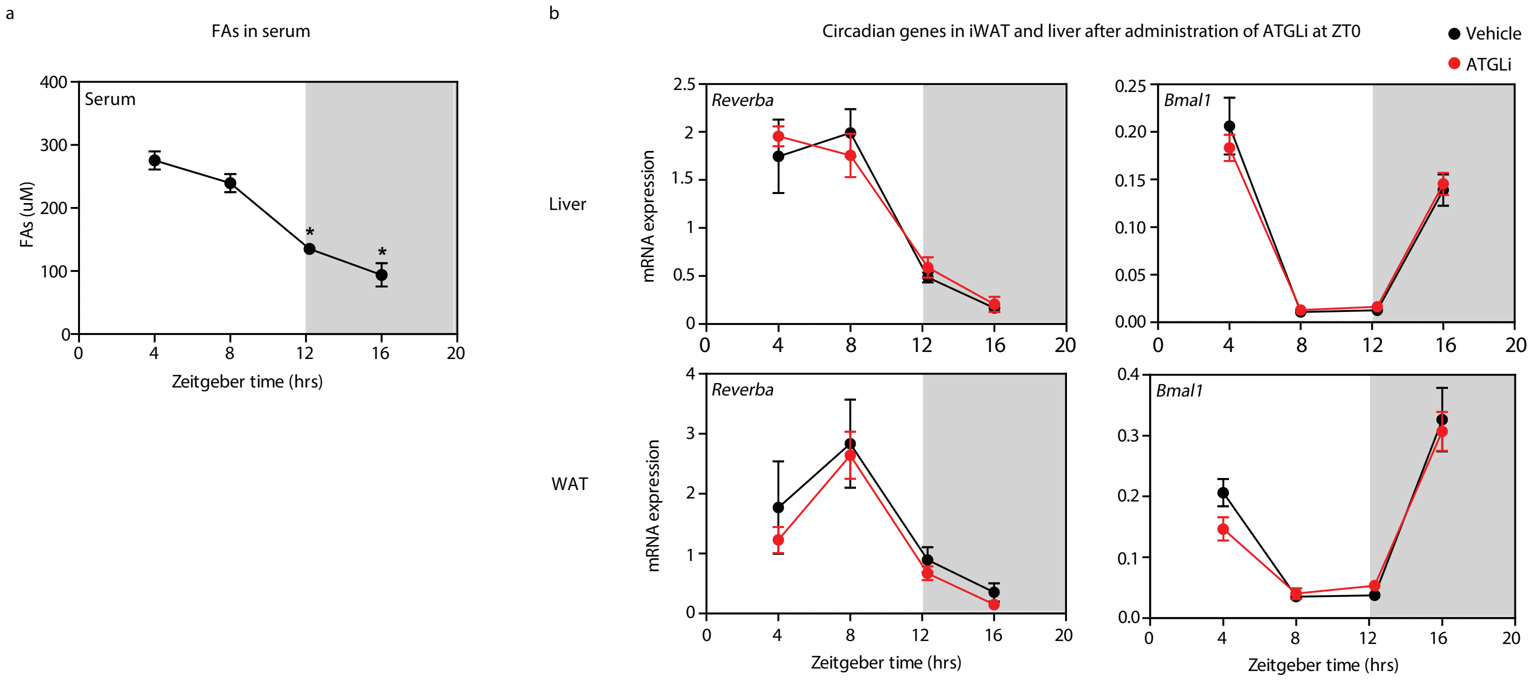
a
FAs in serum
Serum
FAs (uM)
Zeitgeber time (hrs)
b
Circadian genes in iWAT and liver after administration of ATGLi at ZT0
Vehicle
ATGLi
Liver
WAT
Reverba
Bmal1
mRNA expression
Zeitgeber time (hrs)

Supplementary Figure 4: Atglistatin does not override the intrinsic rhythmicity in WAT and liver

(Related to Figure 6)

Male C57BL/6 mice (12 weeks old) received 1.5 mg Atglistatin (red line) or vehicle (black line) by oral gavage at ZT0 and euthanized at ZT4, ZT8, ZT12 and ZT16.

a Serum FA levels in vehicle treated male C57BL/6 mice at ZT4, ZT8, ZT12 and ZT16. The bars represent mean and SEM of 5 mice per time point. **b** mRNA expression of *Reverba* and *Bmal1* in iWAT and liver of male C57BL/6 mice received 1.5 mg Atglistatin (dissolved in oil) by oral gavage at ZT0 and euthanized at ZT4, ZT8, ZT12 and ZT16.

Error bars represent +/- SEM of 5 mice per treatment each time point. Statistical significance was determined by unpaired Student's t-test ($p \leq 0.05$ = *, $p \leq 0.01$ = **, $p \leq 0.001$ = ***).

Supplementary Note: Codes for STAR and MACS2

STAR (ChIP): STAR --genomeLoad LoadAndRemove –genomeDir /data/Genomes/mouse/mm9/star/index101bp/ --runThreadN 8 --readFilesCommand zcat --readFilesIn $FASTQFILE --outSJfilterIntronMaxVsReadN 0 --alignIntronMax 1 --alignSJDBoverhangMin 200 --outFileNamePrefix ${FASTQFILE/.fastq.gz/.star_}

STAT (ATAC): STAR --genomeLoad LoadAndRemove --genomeDir /references/mm9star/index101bp/ --readFilesCommand zcat --runThreadN 16 --readFilesIn $i ${i%_R1_001.fastq.gz}_R2_001.fastq.gz --outSJfilterIntronMaxVsReadN 0 --outFilterMatchNmin 25 --alignIntronMax 1 --alignSJDBoverhangMin 200 --outFileNamePrefix ${i%_R1_001.fastq.gz}

MACS2 (peak calling ChIP): callpeak -t $BAMFILE -c IMPUT.bam -f BAM -g mm –outdir macs2/ -n ${BAMFILE/.bam/.pv-1e-3} -p 1e-3 –to-large
