# Supplementary material for: Lipolysis regulates major transcriptional programs in brown adipocytes
Source: Nat Commun. 2022 Jul 8;13:3956. doi: 10.1038/s41467-022-31525-8 (PMC9270495; doi:10.1038/s41467-022-31525-8)
Supplement: Supplementary file 3 — Description of Additional Supplementary Files [file 41467_2022_31525_MOESM3_ESM.pdf]

Description of Additional Supplementary Files

File Name: Supplementary Data 1
Description: **List of genes with assigned clusters**
List of genes with assigned clusters related to heatmaps in Fig. 1d, Fig. 7a and Supplementary Fig. 1e.

File Name: Supplementary Data 2
Description: **List of transcription factor motifs scoring as medium-to-high confidence causal regulators in response to ISO**
List of inferred causal motifs that are scored as medium to high confidence causal regulators of the transcriptional changes in response to ISO.

File Name: Supplementary Data 3
Description: **List of UPR marker genes**
List of UPR marker genes used for Fig. 5a.
