# Supplementary material for: Lipolysis regulates major transcriptional programs in brown adipocytes
Source: Nat Commun. 2022 Jul 8;13:3956. doi: 10.1038/s41467-022-31525-8 (PMC9270495; doi:10.1038/s41467-022-31525-8)
Supplement: Supplementary file 7 — Reporting Summary [file 41467_2022_31525_MOESM7_ESM.pdf]

nature portfolio

Corresponding author(s): *Susanne Mandrup*

Last updated by author(s): *May 23, 2022*

Reporting Summary

Nature Portfolio wishes to improve the reproducibility of the work that we publish. This form provides structure for consistency and transparency in reporting. For further information on Nature Portfolio policies, see our Editorial Policies and the Editorial Policy Checklist.

Statistics

For all statistical analyses, confirm that the following items are present in the figure legend, table legend, main text, or Methods section.

n/a	Confirmed	
☐	☒	The exact sample size (*n*) for each experimental group/condition, given as a discrete number and unit of measurement
☐	☒	A statement on whether measurements were taken from distinct samples or whether the same sample was measured repeatedly
☐	☒	The statistical test(s) used AND whether they are one- or two-sided<br>*Only common tests should be described solely by name; describe more complex techniques in the Methods section.*
☒	☐	A description of all covariates tested
☐	☒	A description of any assumptions or corrections, such as tests of normality and adjustment for multiple comparisons
☐	☒	A full description of the statistical parameters including central tendency (e.g. means) or other basic estimates (e.g. regression coefficient) AND variation (e.g. standard deviation) or associated estimates of uncertainty (e.g. confidence intervals)
☐	☒	For null hypothesis testing, the test statistic (e.g. *F*, *t*, *r*) with confidence intervals, effect sizes, degrees of freedom and *P* value noted<br>*Give P values as exact values whenever suitable.*
☒	☐	For Bayesian analysis, information on the choice of priors and Markov chain Monte Carlo settings
☒	☐	For hierarchical and complex designs, identification of the appropriate level for tests and full reporting of outcomes
☐	☒	Estimates of effect sizes (e.g. Cohen's *d*, Pearson's *r*), indicating how they were calculated

Our web collection on statistics for biologists contains articles on many of the points above.

Software and code

Policy information about availability of computer code

Data collection	*No software was used for data analysis.*
Data analysis	1) HOMER: http://homer.ucsd.edu/homer/<br>2) STAR: https://github.com/alexdobin/STAR<br>3) DEseq2: https://bioconductor.org/packages/release/bioc/html/DESeq2.html<br>4) IMAGE: https://github.com/JesperGrud/IMAGE<br>5) FastQC: https://www.bioinformatics.babraham.ac.uk/projects/fastqc/<br>4) R Studio: https://www.rstudio.com/<br>5) GraphPad prism: https://www.graphpad.com/scientific-software/prism/<br>6) Biorender: https://biorender.com/<br><br>Codes and scripts used to process and analyze data have been deposited to GitHub: https://github.com/LasseKMarkussen/Lipolysis-regulates-major-transcriptional-programs-in-brown-adipocytes

For manuscripts utilizing custom algorithms or software that are central to the research but not yet described in published literature, software must be made available to editors and reviewers. We strongly encourage code deposition in a community repository (e.g. GitHub). See the Nature Portfolio guidelines for submitting code & software for further information.

Data

Policy information about availability of data

All manuscripts must include a data availability statement. This statement should provide the following information, where applicable:

- Accession codes, unique identifiers, or web links for publicly available datasets
- A description of any restrictions on data availability
- For clinical datasets or third party data, please ensure that the statement adheres to our policy

The datasets generated in this study have been deposited at NCBI GEO under accession code GSE202833 [https://www.ncbi.nlm.nih.gov/geo/query/acc.cgi?acc=GSE202833]. The processed data generated in this study are in Source data and its supplementary materials. Some figures were created using Biorender.

Human research participants

Policy information about studies involving human research participants and Sex and Gender in Research.

Reporting on sex and gender	This study does not contain human research participants.
Population characteristics	This study does not contain population characteristics.
Recruitment	This study does not contain recruitment of human participants.
Ethics oversight	This study does not contain human research participants, therefore, no ethics oversight were included.

Note that full information on the approval of the study protocol must also be provided in the manuscript.

Field-specific reporting

Please select the one below that is the best fit for your research. If you are not sure, read the appropriate sections before making your selection.

- [x] Life sciences
- [ ] Behavioural & social sciences
- [ ] Ecological, evolutionary & environmental sciences

For a reference copy of the document with all sections, see nature.com/documents/nr-reporting-summary-flat.pdf

Life sciences study design

All studies must disclose on these points even when the disclosure is negative.

Sample size	Sample sizes were based on preliminary experiments. All experiments have been performed a minimum of n=2 independent experiments.
Data exclusions	No samples or animals were excluded from the analysis.
Replication	All attempts at replication were successful. To ensure adequate power for statistical analysis at least two independent experiments was performed. For each experiment, the number of independent experiments and statistical analysis performed are noted in the figure legends.
Randomization	Animals were randomized into treatment groups based on body weight to ensure an equal baseline.
Blinding	Blinding was not relevant to this study as all data were derived from absolute quantitative methods without human subjectivity.

Reporting for specific materials, systems and methods

We require information from authors about some types of materials, experimental systems and methods used in many studies. Here, indicate whether each material, system or method listed is relevant to your study. If you are not sure if a list item applies to your research, read the appropriate section before selecting a response.

Materials & experimental systems

n/a	Involved in the study
☐	☒ Antibodies
☐	☒ Eukaryotic cell lines
☒	☐ Palaeontology and archaeology
☐	☒ Animals and other organisms
☒	☐ Clinical data
☒	☐ Dual use research of concern

Methods

n/a	Involved in the study
☐	☒ ChIP-seq
☒	☐ Flow cytometry
☒	☐ MRI-based neuroimaging

Antibodies

Antibodies used	Antibodies to detect total CHOP (#2895, dilution 1:1000), total HSPA5 (#3177, dilution 1:1000) and phosphorylated HSL (Ser-563) (#4139, dilution 1:500) were purchased from Cell Signaling. Antibody to detect H3K27ac (#Ab4729, dilution 2 μg) was purchased from Abcam). Secondary horseradish peroxidase antibodies were from DAKO (#P0447, dilution 1:2000).
Validation	H3K27ac (#Ab4729) antibody has been validated in the ENCODE project: https://www.encodeproject.org/antibodies/ENCAB000BSK/ Total CHOP (#2895), total HSPA5 (#3177) and phosphorylated HSL (Ser-563) (#4139) were all validated by it producing a band of the expected molecular weight for the target protein.

Eukaryotic cell lines

Policy information about cell lines and Sex and Gender in Research

Cell line source(s)	Mouse brown preadipocytes immortalized with SV40 large T antigen, BAT-LgT, were kindly provided by Patrick Seale. Immortalized human brown adipocytes (TERT-hBA) were described Markussen et al., 2017 and Breining et al., 2018.
Authentication	The authentication was tested by differentiation capacity, RNAseq, morphology and metabolic phenotyping.
Mycoplasma contamination	All cell lines were routinely tested for mycoplasma and found negative.
Commonly misidentified lines (See ICLAC register)	No commonly misidentified cell lines were used in this study.

Animals and other research organisms

Policy information about studies involving animals; ARRIVE guidelines recommended for reporting animal research, and Sex and Gender in Research

Laboratory animals	Male C57BL/6N mice (12 weeks old) were housed on a 12:12-h light-dark cycle (lights on at 6 AM, lights off at 6 PM) at room temperature. All mice used in the studies were single housed with ad libitum access to chow diet (Altromin, #30404) and water.
Wild animals	No wild animals were used in this study.
Reporting on sex	Only male mice were used in this study.
Field-collected samples	No field-collected samples were used in this study.
Ethics oversight	All animal studies were performed under Approval #2018-15-0201-01459 from The Danish Animal Experient Inspectorate and complied with the ARRIVE guidelines.

Note that full information on the approval of the study protocol must also be provided in the manuscript.

ChIP-seq

Data deposition

☒ Confirm that both raw and final processed data have been deposited in a public database such as GEO.

☒ Confirm that you have deposited or provided access to graph files (e.g. BED files) for the called peaks.

Data access links<br>*May remain private before publication.*	The datasets generated in this study have been deposited at NCBI GEO under accession code GSE202833 [https://www.ncbi.nlm.nih.gov/geo/query/acc.cgi?acc=GSE202833].
Files in database submission	SM3066_S1_L002_R1_001.fastq.gz<br>SM3066_S1_L002_R2_001.fastq.gz<br>SM3067_S2_L002_R1_001.fastq.gz<br>SM3067_S2_L002_R2_001.fastq.gz

SM3068_S3_L002_R1_001.fastq.gz
SM3068_S3_L002_R2_001.fastq.gz
SM3069_S4_L002_R1_001.fastq.gz
SM3069_S4_L002_R2_001.fastq.gz
SM3070_S5_L002_R1_001.fastq.gz
SM3070_S5_L002_R2_001.fastq.gz
SM3071_S6_L002_R1_001.fastq.gz
SM3071_S6_L002_R2_001.fastq.gz
SM3072_S7_L002_R1_001.fastq.gz
SM3072_S7_L002_R2_001.fastq.gz
SM3073_S8_L002_R1_001.fastq.gz
SM3073_S8_L002_R2_001.fastq.gz
SM3074_S9_L002_R1_001.fastq.gz
SM3074_S9_L002_R2_001.fastq.gz
SM3075_S10_L002_R1_001.fastq.gz
SM3075_S10_L002_R2_001.fastq.gz
SM3076_S11_L002_R1_001.fastq.gz
SM3076_S11_L002_R2_001.fastq.gz
SM3077_S12_L002_R1_001.fastq.gz
SM3077_S12_L002_R2_001.fastq.gz
SM3078_S13_L002_R1_001.fastq.gz
SM3078_S13_L002_R2_001.fastq.gz
SM3079_S14_L002_R1_001.fastq.gz
SM3079_S14_L002_R2_001.fastq.gz
SM3080_S15_L002_R1_001.fastq.gz
SM3080_S15_L002_R2_001.fastq.gz
SM3081_S16_L002_R1_001.fastq.gz
SM3081_S16_L002_R2_001.fastq.gz
SM3082_S17_L002_R1_001.fastq.gz
SM3082_S17_L002_R2_001.fastq.gz
SM3083_S18_L002_R1_001.fastq.gz
SM3083_S18_L002_R2_001.fastq.gz
SM4068_S1_L002_R2_001.fastq.gz
SM4069_S2_L002_R1_001.fastq.gz
SM4069_S2_L002_R2_001.fastq.gz
SM4070_S3_L002_R1_001.fastq.gz
SM4070_S3_L002_R2_001.fastq.gz
SM4071_S4_L002_R1_001.fastq.gz
SM4071_S4_L002_R2_001.fastq.gz
SM4072_S5_L002_R1_001.fastq.gz
SM4072_S5_L002_R2_001.fastq.gz
SM4073_S6_L002_R1_001.fastq.gz
SM4073_S6_L002_R2_001.fastq.gz
SM4074_S7_L002_R1_001.fastq.gz
SM4074_S7_L002_R2_001.fastq.gz
SM4075_S8_L002_R1_001.fastq.gz
SM4075_S8_L002_R2_001.fastq.gz
SM4076_S9_L002_R1_001.fastq.gz
SM4076_S9_L002_R2_001.fastq.gz
SM4077_S10_L002_R1_001.fastq.gz
SM4077_S10_L002_R2_001.fastq.gz
SM4078_S11_L002_R1_001.fastq.gz
SM4078_S11_L002_R2_001.fastq.gz
SM4079_S12_L002_R1_001.fastq.gz
SM4079_S12_L002_R2_001.fastq.gz
SM4080_S13_L002_R1_001.fastq.gz
SM4080_S13_L002_R2_001.fastq.gz
SM4081_S14_L002_R1_001.fastq.gz
SM4081_S14_L002_R2_001.fastq.gz
SM4082_S15_L002_R1_001.fastq.gz
SM4082_S15_L002_R2_001.fastq.gz
SM4083_S16_L002_R1_001.fastq.gz
SM4083_S16_L002_R2_001.fastq.gz
SM4084_S17_L002_R1_001.fastq.gz
SM4084_S17_L002_R2_001.fastq.gz
SM4085_S18_L002_R1_001.fastq.gz
SM4085_S18_L002_R2_001.fastq.gz
7856_S50_L001_R1_001.fastq.gz

7856_S50_L001_R2_001.fastq.gz
7856_S50_L002_R1_001.fastq.gz
7856_S50_L002_R2_001.fastq.gz
7857_S51_L001_R1_001.fastq.gz
7857_S51_L001_R2_001.fastq.gz
7857_S51_L002_R1_001.fastq.gz
7857_S51_L002_R2_001.fastq.gz
7858_S52_L001_R1_001.fastq.gz
7858_S52_L001_R2_001.fastq.gz
7858_S52_L002_R1_001.fastq.gz
7858_S52_L002_R2_001.fastq.gz
7859_S53_L001_R1_001.fastq.gz
7859_S53_L001_R2_001.fastq.gz
7859_S53_L002_R1_001.fastq.gz
7859_S53_L002_R2_001.fastq.gz
7860_S54_L001_R1_001.fastq.gz
7860_S54_L001_R2_001.fastq.gz
7860_S54_L002_R1_001.fastq.gz
7860_S54_L002_R2_001.fastq.gz
7861_S55_L001_R1_001.fastq.gz
7861_S55_L001_R2_001.fastq.gz
7861_S55_L002_R1_001.fastq.gz
7861_S55_L002_R2_001.fastq.gz
SM4004_S1_L001_R1_001.fastq.gz
SM4004_S1_L001_R2_001.fastq.gz
SM4005_S2_L001_R1_001.fastq.gz
SM4005_S2_L001_R2_001.fastq.gz
SM4006_S3_L001_R1_001.fastq.gz
SM4006_S3_L001_R2_001.fastq.gz
SM4007_S4_L001_R1_001.fastq.gz
SM4007_S4_L001_R2_001.fastq.gz
SM4008_S5_L001_R1_001.fastq.gz
SM4008_S5_L001_R2_001.fastq.gz
SM4009_S6_L001_R1_001.fastq.gz
SM4009_S6_L001_R2_001.fastq.gz
SM4010_S7_L001_R1_001.fastq.gz
SM4010_S7_L001_R2_001.fastq.gz
SM4011_S8_L001_R1_001.fastq.gz
SM4011_S8_L001_R2_001.fastq.gz
SM4012_S9_L001_R1_001.fastq.gz
SM4012_S9_L001_R2_001.fastq.gz
SM4013_S10_L001_R1_001.fastq.gz
SM4013_S10_L001_R2_001.fastq.gz
SM4014_S11_L001_R1_001.fastq.gz
SM4014_S11_L001_R2_001.fastq.gz
SM4015_S12_L001_R1_001.fastq.gz
SM4015_S12_L001_R2_001.fastq.gz
SM2848_ATCACG_L001_R1_001.fastq.gz
SM2848_ATCACG_L001_R1_002.fastq.gz
SM2848_ATCACG_L001_R1_003.fastq.gz
SM2848_ATCACG_L002_R1_001.fastq.gz
SM2848_ATCACG_L002_R1_002.fastq.gz
SM2848_ATCACG_L002_R1_003.fastq.gz
SM2849_CGATGT_L001_R1_001.fastq.gz
SM2849_CGATGT_L001_R1_002.fastq.gz
SM2849_CGATGT_L002_R1_001.fastq.gz
SM2849_CGATGT_L002_R1_002.fastq.gz
SM2850_TTAGGC_L001_R1_001.fastq.gz
SM2850_TTAGGC_L001_R1_002.fastq.gz
SM2850_TTAGGC_L002_R1_001.fastq.gz
SM2850_TTAGGC_L002_R1_002.fastq.gz
SM2851_TGACCA_L001_R1_001.fastq.gz
SM2851_TGACCA_L001_R1_002.fastq.gz
SM2851_TGACCA_L002_R1_001.fastq.gz
SM2851_TGACCA_L002_R1_002.fastq.gz
SM2852_ACAGTG_L001_R1_001.fastq.gz
SM2852_ACAGTG_L001_R1_002.fastq.gz
SM2852_ACAGTG_L001_R1_003.fastq.gz

SM2852_ACAGTG_L002_R1_001.fastq.gz
SM2852_ACAGTG_L002_R1_002.fastq.gz
SM2852_ACAGTG_L002_R1_003.fastq.gz
SM2853_GCCAAT_L001_R1_001.fastq.gz
SM2853_GCCAAT_L001_R1_002.fastq.gz
SM2853_GCCAAT_L001_R1_003.fastq.gz
SM2853_GCCAAT_L002_R1_001.fastq.gz
SM2853_GCCAAT_L002_R1_002.fastq.gz
SM2853_GCCAAT_L002_R1_003.fastq.gz
SM2854_CAGATC_L001_R1_001.fastq.gz
SM2854_CAGATC_L001_R1_002.fastq.gz
SM2854_CAGATC_L001_R1_003.fastq.gz
SM2854_CAGATC_L001_R1_004.fastq.gz
SM2854_CAGATC_L002_R1_001.fastq.gz
SM2854_CAGATC_L002_R1_002.fastq.gz
SM2854_CAGATC_L002_R1_003.fastq.gz
SM2854_CAGATC_L002_R1_004.fastq.gz
SM2855_ACTTGA_L001_R1_001.fastq.gz
SM2855_ACTTGA_L001_R1_002.fastq.gz
SM2855_ACTTGA_L001_R1_003.fastq.gz
SM2855_ACTTGA_L001_R1_004.fastq.gz
SM2855_ACTTGA_L002_R1_001.fastq.gz
SM2855_ACTTGA_L002_R1_002.fastq.gz
SM2855_ACTTGA_L002_R1_003.fastq.gz
SM2855_ACTTGA_L002_R1_004.fastq.gz
SM2856_GATCAG_L001_R1_001.fastq.gz
SM2856_GATCAG_L001_R1_002.fastq.gz
SM2856_GATCAG_L001_R1_003.fastq.gz
SM2856_GATCAG_L002_R1_001.fastq.gz
SM2856_GATCAG_L002_R1_002.fastq.gz
SM2856_GATCAG_L002_R1_003.fastq.gz
SM2857_TAGCTT_L001_R1_001.fastq.gz
SM2857_TAGCTT_L001_R1_002.fastq.gz
SM2857_TAGCTT_L001_R1_003.fastq.gz
SM2857_TAGCTT_L001_R1_004.fastq.gz
SM2857_TAGCTT_L001_R1_005.fastq.gz
SM2857_TAGCTT_L002_R1_001.fastq.gz
SM2857_TAGCTT_L002_R1_002.fastq.gz
SM2857_TAGCTT_L002_R1_003.fastq.gz
SM2857_TAGCTT_L002_R1_004.fastq.gz
SM2857_TAGCTT_L002_R1_005.fastq.gz
SM2858_GGCTAC_L001_R1_001.fastq.gz
SM2858_GGCTAC_L001_R1_002.fastq.gz
SM2858_GGCTAC_L001_R1_003.fastq.gz
SM2858_GGCTAC_L002_R1_001.fastq.gz
SM2858_GGCTAC_L002_R1_002.fastq.gz
SM2858_GGCTAC_L002_R1_003.fastq.gz
SM2859_CTTGTA_L001_R1_001.fastq.gz
SM2859_CTTGTA_L001_R1_002.fastq.gz
SM2859_CTTGTA_L001_R1_003.fastq.gz
SM2859_CTTGTA_L002_R1_001.fastq.gz
SM2859_CTTGTA_L002_R1_002.fastq.gz
SM2859_CTTGTA_L002_R1_003.fastq.gz
SM2860_AGTCAA_L001_R1_001.fastq.gz
SM2860_AGTCAA_L001_R1_002.fastq.gz
SM2860_AGTCAA_L001_R1_003.fastq.gz
SM2860_AGTCAA_L002_R1_001.fastq.gz
SM2860_AGTCAA_L002_R1_002.fastq.gz
SM2860_AGTCAA_L002_R1_003.fastq.gz
SM2861_AGTTCC_L001_R1_001.fastq.gz
SM2861_AGTTCC_L001_R1_002.fastq.gz
SM2861_AGTTCC_L001_R1_003.fastq.gz
SM2861_AGTTCC_L001_R1_004.fastq.gz
SM2861_AGTTCC_L002_R1_001.fastq.gz
SM2861_AGTTCC_L002_R1_002.fastq.gz
SM2861_AGTTCC_L002_R1_003.fastq.gz
SM2861_AGTTCC_L002_R1_004.fastq.gz
SM2862_ATGTCA_L001_R1_001.fastq.gz

SM2862_ATGTCA_L001_R1_002.fastq.gz
SM2862_ATGTCA_L001_R1_003.fastq.gz
SM2862_ATGTCA_L002_R1_001.fastq.gz
SM2862_ATGTCA_L002_R1_002.fastq.gz
SM2862_ATGTCA_L002_R1_003.fastq.gz
SM2863_CCGTCC_L001_R1_001.fastq.gz
SM2863_CCGTCC_L001_R1_002.fastq.gz
SM2863_CCGTCC_L001_R1_003.fastq.gz
SM2863_CCGTCC_L002_R1_001.fastq.gz
SM2863_CCGTCC_L002_R1_002.fastq.gz
SM2863_CCGTCC_L002_R1_003.fastq.gz
SM2864_GTCCGC_L001_R1_001.fastq.gz
SM2864_GTCCGC_L001_R1_002.fastq.gz
SM2864_GTCCGC_L001_R1_003.fastq.gz
SM2864_GTCCGC_L002_R1_001.fastq.gz
SM2864_GTCCGC_L002_R1_002.fastq.gz
SM2864_GTCCGC_L002_R1_003.fastq.gz
SM2865_GTGAAA_L001_R1_001.fastq.gz
SM2865_GTGAAA_L001_R1_002.fastq.gz
SM2865_GTGAAA_L001_R1_003.fastq.gz
SM2865_GTGAAA_L002_R1_001.fastq.gz
SM2865_GTGAAA_L002_R1_002.fastq.gz
SM2865_GTGAAA_L002_R1_003.fastq.gz
Counts_Dox_exp.txt
Raw_Count_Matrix.txt
Lipolysis_DK.txt
Triascin.txt

Genome browser session (e.g. UCSC)

No longer applicable.

Methodology

Replicates

ChIP-seq data were generated from two independent experiments of brown adipocytes. STAR derived .bam files were used as input for MACS2 at a low threshold. MACS2 peak calling was done on a pooled library of both replicates for each condition. Input DNA was used as background for peak detection. Peaks with an IDR ≤ 0.05 were kept and merged using HOMER (v4.10) mergePeaks function with distance -d given.

Sequencing depth

Uniquely mapped reads >75% for all samples. Sequencing depth was 15 million reads per sample.

Antibodies

Rabbit polyclonal H2K27Ac from Abcam #Ab4729, dilution 2 µg. RRID: AB_2118291.

Peak calling parameters

STAR derived .bam files were used as input for MACS2 at a low threshold. MACS2 peak calling was done on a pooled library of both replicates for each condition. Input DNA was used as background for peak detection. Peaks with an IDR ≤ 0.05 were kept and merged using HOMER (v4.10) mergePeaks function with distance -d given. ChIP-seq data were generated from two independent experiments.

STAR (ChIP): STAR --genomeLoad LoadAndRemove –genomeDir /data/Genomes/mouse/mm9/star/index101bp/ --runThreadN 8 --readFilesCommand zcat --readFilesIn $FASTQFILE --outSJfilterIntronMaxVsReadN 0 --alignIntronMax 1 --alignSJDBoverhangMin 200 --outFileNamePrefix ${FASTQFILE/.fastq.gz/.star_}

MACS2 (peak calling ChIP): callpeak -t $BAMFILE -c IMPUT.bam -f BAM -g mm –outdir macs2/ -n ${BAMFILE/.bam/.pv-1e-3} -p 1e-3 –to-large

Data quality

Uniquely mapped reads >75% for all samples and reads were identified on all chromosomes. Peak quality was ensured by the Idr ENCODE pipeline. Only one read per position was per length was allowed to ensure removal of potential PCR duplicates.

Software

Sequencing quality was checked using FastQC (https://www.bioinformatics.babraham.ac.uk/projects/fastqc/). Reads were aligned using STAR (https://github.com/alexdobin/STAR). Bam files were used peakcalling using MACS2 (https://github.com/topics/macs2). Merging and quantification of peaks was done using HOMER (http://homer.ucsd.edu/homer/).
